# CHAIMELEON Project: Creation of a Pan-European Repository of Health Imaging Data for the Development of AI-Powered Cancer Management Tools

**DOI:** 10.3389/fonc.2022.742701

**Published:** 2022-02-24

**Authors:** Luis Martí Bonmatí, Ana Miguel, Amelia Suárez, Mario Aznar, Jean Paul Beregi, Laure Fournier, Emanuele Neri, Andrea Laghi, Manuela França, Francesco Sardanelli, Tobias Penzkofer, Phillipe Lambin, Ignacio Blanquer, Marion I. Menzel, Karine Seymour, Sergio Figueiras, Katharina Krischak, Ricard Martínez, Yisroel Mirsky, Guang Yang, Ángel Alberich-Bayarri

**Affiliations:** ^1^ Medical Imaging Department, La Fe University and Polytechnic Hospital & Biomedical Imaging Research Group Grupo de Investigación Biomédica en Imagen (GIBI2^30^) at La Fe University and Polytechnic Hospital and Health Research Institute, Valencia, Spain; ^2^ Matical Innovation SL, Madrid, Spain; ^3^ Collège des enseignants en radiologie de France, Paris, France; ^4^ Diagnostic Radiology 3, Department of Translational Research, University of Pisa, Pisa, Italy; ^5^ Medicina Traslazionale e Oncologia, Sant Andrea Sapienza Rome, Rome, Italy; ^6^ Department of Radiology, Centro Hospitalar Universitário do Porto, Porto, Portugal; ^7^ Servizio di Diagnostica per Immagini, “Istituto di Ricovero e Cura a Carattere Scientifico (IRCCS) Policlinico San Donato, Milanese, Italy; ^8^ Department of Radiology, CHARITÉ-Universitätsmedizin Berlin, Berlin, Germany; ^9^ Department of Precision Medicine, Maastricht University, Maastricht, Netherlands; ^10^ Computing Science Department, Universitat Politècnica de València, València, Spain; ^11^ GE Healthcare, München, Germany; ^12^ Department of Physics, Technical University of Munich, Garching, Germany; ^13^ Medexprim, Labège, France; ^14^ Bahia Software S.L.U., Coruña, Spain; ^15^ European Institute for Biomedical Imaging Research, EIBIR gemeinnützige GmbH, Vienna, Austria; ^16^ Departamento de Derecho Constitucional, Ciencia Política y Administración, Universitat de València, València, Spain; ^17^ Software and Information Systems Engineering, Ben-Gurion University of the Negev, Beer Sheva, Israel; ^18^ National Heart and Lung Institute, Imperial College London, London, United Kingdom; ^19^ Quantitative Imaging Biomarkers in Medicine, QUIBIM SL, Valencia, Spain

**Keywords:** radiology, artificial intelligence-AI, cancer imaging, cancer management, quantitative imaging biomarkers, image harmonization

## Abstract

The CHAIMELEON project aims to set up a pan-European repository of health imaging data, tools and methodologies, with the ambition to set a standard and provide resources for future AI experimentation for cancer management. The project is a 4 year long, EU-funded project tackling some of the most ambitious research in the fields of biomedical imaging, artificial intelligence and cancer treatment, addressing the four types of cancer that currently have the highest prevalence worldwide: lung, breast, prostate and colorectal. To allow this, clinical partners and external collaborators will populate the repository with multimodality (MR, CT, PET/CT) imaging and related clinical data. Subsequently, AI developers will enable a multimodal analytical data engine facilitating the interpretation, extraction and exploitation of the information stored at the repository. The development and implementation of AI-powered pipelines will enable advancement towards automating data deidentification, curation, annotation, integrity securing and image harmonization. By the end of the project, the usability and performance of the repository as a tool fostering AI experimentation will be technically validated, including a validation subphase by world-class European AI developers, participating in Open Challenges to the AI Community. Upon successful validation of the repository, a set of selected AI tools will undergo early *in-silico* validation in observational clinical studies coordinated by leading experts in the partner hospitals. Tool performance will be assessed, including external independent validation on hallmark clinical decisions in response to some of the currently most important clinical end points in cancer. The project brings together a consortium of 18 European partners including hospitals, universities, R&D centers and private research companies, constituting an ecosystem of infrastructures, biobanks, AI*/in-silico* experimentation and cloud computing technologies in oncology.

## Introduction

The use of Artificial Intelligence (AI) on health data is generating promising tools to assist clinicians in cancer management, as an increasing number of health imaging-based AI approaches are proving to have vast potential to become useful clinical tools in different areas of application (1). These include recurrence and survival prediction using multidimensional heterogeneous data (2) prediction of tumor molecular features and association with tumor spread (3, 4), stratification of patients based on risk (5), and prediction of treatment response (6) among many others.

Despite these major advancements, the development of imaging-based AI tools relies on the availability of large, quality-controlled datasets (7), which currently still remains a major challenge. The generation of these imaging biobanks is a resource-intensive endeavor, facing multiple technical and operational difficulties such as image and data harmonization, data curation and annotation, image pre-processing and annotation, as well as various legal and ethical restrictions (8–13). As a result, the quantity, quality, and representativeness of datasets still remain major limiting factors in the development of predictive cancer management tools.

Despite these limitations, several health imaging repositories have been created to date (14–16), such as the Cancer Imaging Archive (TCIA) which is one of the most renowned amongst those focusing on cancer imaging (17). Albeit of huge potential, the vast majority of these repositories have been created as stand-alone entities, being currently not in a position to become interoperable with similar existing initiatives. As such, the need for the creation of a fully FAIR (Findable, Accessible, Interoperable, Reusable), GDPR compliant, European imaging repository still stands (18).

To address the lack of data availability as well as the interoperability limitation of currently existing initiatives, the CHAIMELEON project aims to set-up and populate a cancer imaging repository facilitating access to large, high-quality sets of anonymized data. This will be achieved through the creation of a distributed data repository that will be made interoperable with other existing repositories and biobanks, enabling secure share and reuse of data as an intuitive sustainable single-access point resource for the community of developers working on AI-powered cancer management solutions. The repository will use a controlled access policy, whereby registered users will have access to datasets upon acceptance of the terms and conditions of use. These conditions, albeit still under definition by the governance bodies, will include the contracting of non-identification commitments as well as others related to the purpose of use of the data. Since imaging datasets contain images acquired at different centers with different scanners (cross-vendor/cross-institution datasets), quantitative image features, parameters, values and ranges extracted from images acquired at one center may not be reproducible from once center to another. This is due to a lack of consistency of medical images, as they generate from different equipment vendors, models and software versions. To ensure the reproducibility of quantitative imaging biomarkers (QIB) and allow scientific reuse of retrospective imaging data from multicenter acquisitions, CHAIMELEON has set the development and testing of imaging data harmonization protocols as one of its main objectives. Different harmonization approaches have been proposed, including a disruptive one for the generation of synthetic images adjusted to a common harmonization framework, ensuring that the authenticity and integrity of each synthetic coherent image is properly secured.

**Figure 1:**
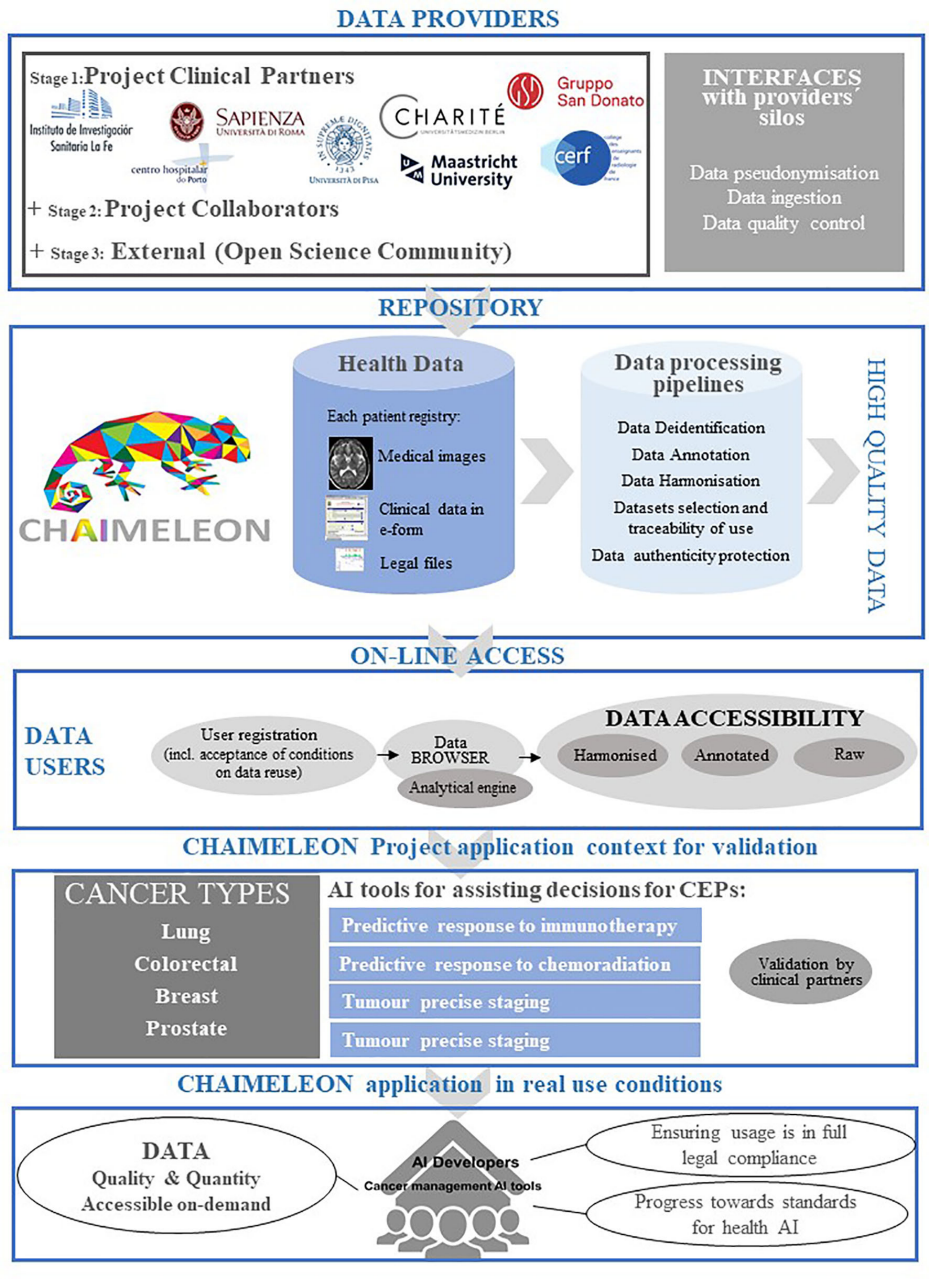
Project overview.

The project will involve the setup of the IT infrastructure, the creation of protocols for legal compliance, the development of tools for agile data ingestion and curation and processing pipelines for imaging data annotation and harmonization, as well as methodologies and tools for enhanced interpretability of AI models among others. Since the repository will be targeted to AI developers as end-users, it will not be designed as a simple data warehouse but as a complete AI-powered solution that will provide integrated quality data. To do so, AI-powered pipelines for data annotation, multicentric data harmonization, integrity securing of synthetic AI-generated images and clinical decision prediction will be implemented, aiming to enhance the interpretability of the tested AI models. An overview of the project execution steps is summarized in **Figure 1**.

Ethics, integrity and compliance with data protection regulatory frameworks will be integral and critical to the project, guaranteeing regulatory compliance in all aspects of the work performed both at the technical and clinical level. To ensure this, all project actions will be carefully guided and supervised by legal experts on data privacy.

The successful execution of this project will make multiple resources available to the research community. These will include data infrastructures, legal protocols, new AI models and tools, and methodologies for performance validation and enhanced interpretability of AI solutions. All these resources will be ultimately validated in the context of lung, breast, colorectal and prostate cancer. Given that these are such complex and heterogeneous diseases, this will allow to demonstrate the versatility and scalability of the repository and tools across a range of heterogeneous cancer applications. The final aim is to design the repository as a transversal resource with the required versatility and scalability to incorporate data for other types of cancer and imaging modalities in the near future.

## Materials and Methods

### Project Consortium

To bring this project to fruition, an interdisciplinary consortium was recruited, bringing together experts in the fields of IT systems, automated health data management, data privacy and legal compliance, and researchers with experience in the design, set-up and management of imaging repositories and radiomic features. The CHAIMELEON consortium involves 18 partners from 9 European countries (Spain, Germany, France, Austria, United Kingdom, Israel, Italy, The Netherlands and Portugal) constituting a pan-European ecosystem of knowledge, infrastructures, biobanks and technologies in oncology, AI/*In-silico* experimentation and cloud computing. The partner institutions include 9 hospitals, 3 universities, and 6 R&D centers and private companies. An advisory board consisting of a recognized group of experts in the fields of oncology and AI applied to cancer management has been designated to give general advice and guidance to the consortium.

### Project Timeline and Management Strategy

The CHAIMELEON project is a 4-year long, EU funded project that started in September 2020 and will finalize in August 2024. Currently in its second year of execution, the project results already include a first complete version of the repository design, a selected set of standards to be used to ensure the repository’s interoperability and the first proposal for its legal operating model among others. To ensure its correct implementation, the project has set 13 different milestones to be met over its entire duration. A final, populated version of the repository is expected to be available to the public on project month 34 (June 2023). A summary of relevant project milestones indicating dataset availability of the repository can be found in **Table 1**.

**Table 1:** Project milestones.

Milestone ID	Description	Due date (months)
M1	Initial repository design available for regulatory clearance. Repository’s legal operational model established.	12
M2	Start of data collection at data provider sites, with clearance for data to be incorporated into the CHAIMELEON repository.	13
M3	Completion of the repository design phase and the verification of the repository’s compliance with GDPR.	18
M4	First repository prototype released, fully interfaced with data provider sites	24
M5	Start of the repository’s technical validation phase Stage 1 – Internal by project partners	30
M6	Start of the repository’s technical validation phase Stage 2 – External validation *via* open challenges to the AI community	31
M7	End of the repository’s technical validation phase Stage 1 – Internal validation completed and documented	34
M8	Execution of the repository’s technical validation phase Stage 2 – External validation *via* open challenges	34
M9	Start of the repository’s data expansion stage – addition of new datasets provided by external collaborators. Legal clearance and IT interfacing with selected centers.	37
M10	End of the repository’s technical validation phase Stage 2 – External validation *via* open challenges	38
M11	Start of the clinical validation phase - observational studies for AI-based solutions developed/refined using the repository start	41
M12	End of the clinical validation phase	46
M13	Assessment of observational studies finalized.	48

### Distributed Architecture and IT Infrastructure of the Repository

To facilitate its scalability and promote cooperative work with the rest of the scientific community, the repository is cloud-based and built upon open standards. Likewise, the use of free, open-access services has been prioritized, to keep maintenance and running costs to a minimum while implementing a robust software infrastructure. In particular, CHAIMELEON uses European Open Science Cloud (EOSC) services to join the projects currently contributing to this initiative. **Table 2** summarizes CHAIMELEON’s main design and infrastructure features.

**Table 2:** Summary of CHAIMELEON’s main design and infrastructure features.

Feature	Description
Distributed infrastructure	In the first phase of the project, data will be centralized, after it has been collected, curated and anonymized by a set of tools deployed locally. In the second phase of the project, we will explore a distributed architecture, where the architecture will be composed of a central index and multiple physical repositories (local indexes), which may be either regional, national or hospital-based data warehouses connected to the hospital’s PACS and EHR/RIS. Repositories will be connected using encrypted communications and standards for interoperability, such as DICOM-TLS or DICOM web. Federated Learning approaches and distributed data exploration solutions will be explored.
Single-entry point for pan- European users	CHAIMELEON will be designed to facilitate AI developers access to any relevant curated datasets, independently of their origin.
Publicly available, upon user registration	The registration process will include requirements for the researchers to sign acceptance of the conditions of use and access to the data. These will include commitments related to the purposes of data use and contracting of non-identification commitments.
Types of roles	Different entities and physical persons under different roles will be key parties to the repository, including data providers, entities providing infrastructure or services (primary data users), and researchers willing to access data for research purposes (data users). Roles will be carefully defined and assigned the applicable rights and obligations.
Powered with automatic tools, human refined	The latest machine learning advancements on data ingestion, curation, quality control, annotation, segmentation and harmonization will be incorporated into CHAIMELEON. During the project execution, extensive human resources will be devoted to the supervision and refinement of the automation tools. As technologies evolve, the repository will steadily progress towards less human supervision and more automated processes.
Pseudonymized and anonymized data	The Repository will have two levels of de-identification. The first one will be pseudonymization at local premises, in order to preserve traceability and enable potential linkage to other biobanks (e.g., Pathological or genetic). The second, at the central repository level, will be complete anonymization, meaning the data will no longer be identifiable, even indirectly.

**Figure 2:**
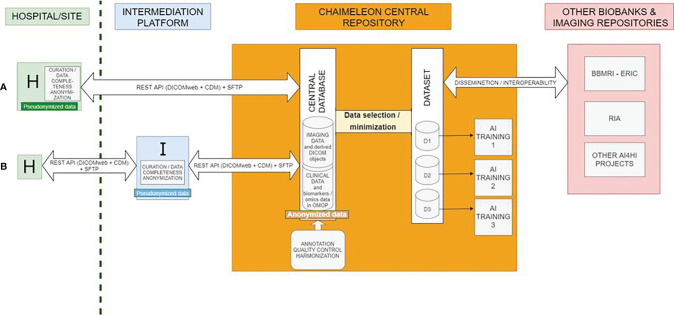
Distributed architecture and IT infrastructure. **(A)** and **(B)** refer to two different types of hospitals based on their capacity to curate, complete, and anonymize data prior to their ingestion into the central repository. **(A)** Data processing is done on site. **(B)** Data processing is done via an intermediation platform.

The CHAIMELEON repository architecture has been set as a hybrid type of architecture whereby local data warehouses and tools deployed within hospitals streamline the process of data collection and curation, while a central repository allows (1) management and annotation of anonymized data (2) AI model training, and (3) use of data processing pipelines. Local tools deployed at hospitals are being built on the *Medexprim Suite™* framework to allow cohort selection, image extraction, collection, extraction and mapping of clinical data, as well as data curation, quality control, anonymization and transfer to the central repository (**Figure 2**). This set of tools is currently undergoing constant customization in order to adjust to the specific IT environment of each data provider site.

**Figure 3:**
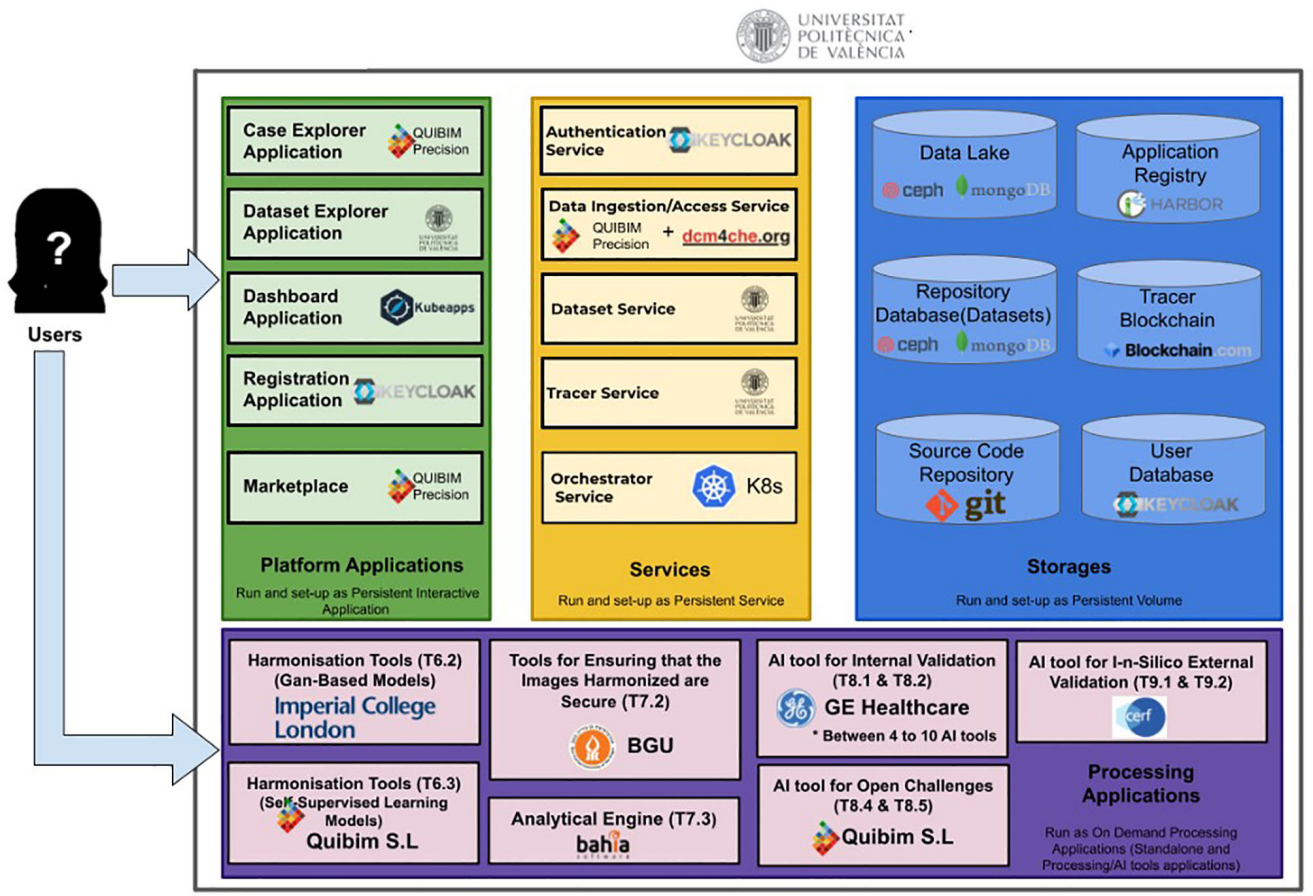
High-level architecture of the central repository and technologies used.

The storage and processing platform (central repository) uses a set of horizontal technologies that enable the distributed storage of medical images and associated clinical data, along with a processing environment where different applications can run. In particular, the storage has been implemented through CEPH (19) running on an on-premise cloud infrastructure implemented through OpenStack (20). The application and services run embedded on Docker (21) containers orchestrated by a Kubernetes (22) platform. Images are stored as DICOM files on a filesystem hierarchy, using access control lists to manage authorization. Clinical data is stored on a Mongo DB (23) through standardized e-forms and authentication is based on OpenID (24). A set of services has been developed to map access permissions, mount selected data on virtual workstation consoles and persist access and data processing actions on a Blockchain registry. Some data processing applications including one for image harmonization are currently being developed by project partners as one of the main challenges to this project. The overall platform architecture and list of processing applications is illustrated in **Figure 3**. In line with making the repository data FAIR, data ingestion processes ensure the incorporation of relevant information and make sure datasets are searchable by different criteria such as type of disease, imaging modality, or patient´s gender and age. Registered users (data requesters adhering to the data usage policy/license) will have on-line, controlled access to anonymized data only. Accessibility to pseudonymized data, on the other hand, will be limited to authorized repository managers locally in order to enable linkage to other related biobanks and the use of standards for metadata exchange and annotation. Data usage policies and licenses are currently being defined for users to commit to the reuse data for research purposes mainly.

The repository design has been built under the principles of legal compliance and privacy and security by design and by default. To do so, the consortium’s data protection officer has performed a risk analysis and data protection impact assessment, identifying security, ethics, and regulatory compliance risks, as well as those associated with the re-identification of patients and the potential impact of the use of AI.

The long-term sustainability of the repository is being promoted by design, as it is being established as a controlled access cloud-based repository hosting reusable methodologies and protocols which will be interoperable with similar existing initiatives. Other means for the continuity of the repository will be assessed over the project lifetime, including creating synergies with new repositories and biobanks and incorporating data for other types of cancer and imaging modalities. Methodologies and protocols, including image processing tools, automatic annotation pipelines, and tools promoting interpretability of the AI models will be made available for reuse by similar research initiatives in the health imaging community.

### Repository Population and Image Harmonization Strategies

The data to be made accessible in the repository will include imaging data (complete radiological studies for a given case) in DICOM format, linked to the correspondent e-form including relevant clinical features on the patient´s profile (age, gender, ethnicity, symptoms, comorbidities, etc.), tumor data (pathological, molecular and genetic), treatment details and outcome.

These data will be provided by the clinical partner institutions in the consortium which will populate the repository with retrospective cases during the project lifetime over two data recruitment periods. All cases will correspond to real world data, meaning the study subjects will be patients diagnosed in the participant hospitals for the 4 targeted types of cancer, collected through the routine delivery of healthcare with no enrolment conditions. Local imaging protocols will be used all over the data recruitment period. All cases included will be fully closed cases, that is, those for which all the required data, as specified in the project requirements, are already available for a given patient. More specifically, both clinical and imaging data will be collected over the period comprised between the date of diagnosis and a 12 or 24 month follow up, depending on the type of cancer.

It is estimated that de-identified medical imaging data and related clinical data for a total of 11.500 cases of lung cancer, 6.000 of breast cancer, 6.000 of colorectal cancer and 10.000 of prostate cancer approximately will be accessible from the repository. **Table 3** shows a summary of the type of data expected to be accessible in CHAIMELEON per type of cancer.

**Table 3:** Types of datasets to be accessible from the CHAIMELEON repository.

Type of cancer	Imaging Data	Estimated number of cases	
		Training phase	Validation phase
**Lung cancer**	CT/PET/CT	7000	4500
**Breast cancer**	Mammography, Digital breast tomosynthesis, Ultrasound and MRI	3500	2500
**Colorectal cancer**			
Colon	CT	2334	1667
Rectum	MRI	1167	833
**Prostate cancer**	MRI	6000	4000

To further accelerate the Repository´s population and to evidence its scalability and adaptability, during project Stage 2 (years 3 and 4) external collaborator hospitals from 5 additional countries will join the data provision efforts. These independent cases will be used for the external validation of the AI tools within the repository architecture. With both internal and external cases incorporated, the total size forecasted for the repository by project end is of nearly 40.000 cases, corresponding to approximately 20 million images (25).

To address the data inhomogeneity derived from the multiple sources the images will originate from, computational data harmonization methods such as self-supervised learning and GAN based models will be developed, in an aim to merge the data from the different sources into a single coherent data set by modifying data formats, terminologies, and measurement units (12, 13, 26). The project will consider reusing the results of the Imaging Biomarker Standardisation Initiative (27). Computational data harmonization methods, such as ComBat including its variations (BM-ComBat and QN-ComBat), and Distance-Weighted Discrimination, will also be explored (11).

Lastly, and so as to ensure that CHAIMELEON will offer only high-quality datasets, a data submission process will be implemented, involving the performance of quality assurance tests and the assignment of data set quality scores upon data submission onto the central repository. A detailed description of the quality check process is described in the **Supplementary Material**.

### Data Models and FAIR Principles

CHAIMELEON’s approach to image metadata is oriented at enabling data interoperability following FAIR data principles. As such, the data model to be implemented will refer to the current DICOM-MIABIS joint model (28), which proposes a first integration of the international DICOM standards into the MIABIS (Minimum Information About Biobank Data Sharing) core model. While MIABIS aims to standardize data elements used to describe biobanks and samples, the DICOM fields will be used to describe heterogeneous information across datasets, such as imaging protocols, modalities, sequences, scanners, and labels. Complete DICOM information (excluding patient information) will be available for further description of imaging collections. Therefore, the model will provide for the inclusion of metadata relating to the acquisition parameters, MRI and CT. Pseudo-anonymization processes, where applicable, will be programmed for the conservation of essential metadata such as patient preparation protocol. In this manner, this project further highlights the current need of datasheets for datasets (29)

Regarding the Common Data Model (CDM) to be used for clinical data across clinical centers, the use of the OMOP (Observational Medical Outcomes Partnership) CDM (30) was agreed upon, which is based on standard ontologies such as SNOMED CT] (31) or ICD-10 (32). As such, the local clinical data warehouse deployed at each hospital is built using the OMOP CDM, and the set of necessary clinical variable terms to collect has been standardized according to this standard. As of project month 16, a total of 930 terms (97%) have been successfully adapted to match the standard vocabularies with currently available concepts. The CHAIMELEON project is committed to contributing to the evolution of standards rather than developing proprietary formats. For this reason, an upgrade of the OMOP CDM has been suggested to include the 24 remaining terms (3%) given their relevance in current clinical practice.

As curated, annotated, and enriched datasets are being constituted, the MIABIS model will be used to label these collections and promote their reuse. Findability, on the other hand, will be provided through the use of persistent identifiers that will be assigned to the datasets and persisted on the Blockchain. Accessibility will be ensured through the use of an open Application Programming Interface (API) to query the dataset service. Reusability will then be enhanced by the provision of virtual processing environments.

### Development of AI Tools for Cancer Management

The project will contribute to the development, refining, testing and early clinical validation of AI tools targeted to reproducibly assist clinicians in the precise estimation of some of the currently most relevant Clinical End Points (CEPs) in cancer (**Table 4**) (33). The tools include image preparation and harmonization, tissue segmentation, radiomics data extraction, treatment allocation and prognosis prediction (7, 34–37). Once these are tested, Open Challenges will be organized promoting other world-class developers to use CHAIMELEON resources to train their own models.

**Table 4:** Clinical end points to be addressed in CHAIMELEON for the four targeted types of cancer.

Type of Cancer	Current therapies	CEPs
Lung	Immunotherapy	Predicting patients with a positive response to immunotherapy
Colorectal	Surgery/neoadjuvant chemotherapy	(Rectal cancer) Prediction of patients with a positive response to chemoradiation and classification in different treatment response sub-groups.
		(Colon cancer) Identification of patients at higher risk of distant metastases at an early timepoint.
Breast	Surgery, radiation and systemic therapy	Diagnostic performance and cancer staging.
Prostate	Wide range due to heterogeneity	Early Staging/Grading

This project will follow a methodological approach of continuous learning, allowing a smooth update of the models including new data annotations and training to progressively improve performance over time. It is expected that this continuous learning will provide the repository infrastructure with large scalability in terms of algorithm performance and management of datasets.

Although many AI models that predict factors related to both disease phenotyping and treatment effects have recently been published (38–45), important challenges remain in the standardization of the criteria for evaluation of model performance, reproducibility and clinical utility. The principal challenge remains the optimal collection and integration of diverse multimodal data sources in such quantitative manner that delivers unambiguous clinical predictions. In CHAIMELEON, we aim to undertake ground-breaking research on the AI space leading to a new paradigm in the investigation of imaging biomarkers at multi-center studies and clinical trials, overcoming the problem of reproducibility in QIBs.

The cancer management solutions to be developed in the context of this project include those assisting radiologists in image processing and analysis, impacting diagnosis and follow up capabilities, helping predict tumor behavior, as well as aiding patient’s stratification, therapy allocation and tumor response to treatment.

### Technical and Clinical Validation of AI Solutions

By the end of the project, the performance of the repository as tool fostering AI experimentation will be validated. This technical validation phase will assess the usefulness of the repository for accelerating experimentation of AI solutions and contributing to better training and testing of the AI models. This process will occur in two subsequent steps. First, AI developers of the consortium will undertake training and testing of a selected set of their proprietary AI tools using the data provided by consortium partners. Secondly, an external validation subphase will be done by other world-class European AI developers, initiative that will be articulated *via* Open Challenges to the AI community.

Lastly, a clinical validation phase will assess how the technically validated tools can assist clinicians in addressing the selected CEPs for lung, breast, colorectal and prostate cancer. To do so, observational *In-silico* studies will be designed by cancer experts to assess the capacity of these tools to aid clinical decision-making in cancer management in terms of diagnosis, prognosis, treatment selection and treatment follow-up. Cases provided by external collaborator centers will be used for this clinical validation phase to ensure the reproducibility of the results on real world cases.

## Expected Results

The CHAIMELEON repository along with its related AI-powered tools are being designed to impact the management of the four most prevalent types of cancer worldwide. Due to the social and economic burden these imply, we expect the outcome of this project to have an EU-wide impact both at the social and economic level. Upon successful validation on how the proposed AI tools can assist clinicians in daily decision making, we expect the repository infrastructure, legal operational model, analysis tools and web-based user interfaces to have the potential to be adapted to the management of other types of cancer (46).

The project will contribute to the current state of the art of AI for health imaging by defining a framework for legitimate access to anonymized imaging and related clinical data provided by hospitals in different European nations, making these more openly accessible across the EU for secondary use in research. From image acquisition to image evaluation and QIB reporting, our work is aligned with exciting research on the use of AI for improving image quality and interpretation. At the technical level, we hope to contribute to the advancement in robustness of AI systems against malicious attacks, interpretability of AI-based models, and validation of AI tools in clinical observational studies. We also aim to impact the fields of standardization of radiological procedures for image acquisition and analysis, as well as harmonization for extraction of reproducible imaging biomarkers.

From a legal perspective, this project will contribute to the creation of ethical standards for the use of health imaging data in the context of AI tool development. All in all, we expect CHAIMELEON to generate resources that facilitate faster and more successful development of AI-based solutions for cancer management, while promoting actions to foster the evolution of European laws in the reuse of health data for research purposes (47). By doing so, we expect to have a major mid to long-term impact in the European health imaging sector, increasing trust in AI solutions amongst healthcare professionals, patients, and stakeholders in both industry and academia.

## Discussion

The EU-funded CHAIMELEON project aims to set up one of the most ambitious health imaging repositories across the EU, contributing to major advancements in the field of cancer management at a global scale. Once implemented, it will have the potential to generate innovative products and services beyond the direct outcomes planned for the project. The developed methodologies and protocols will pave the way for the use of this type of repository, not just in other fields of biomedical research but also in any other disciplines where public interest is the main driver.

Upon successful demonstration of the disruptive analysis approaches used in this project, such as those related to the improvement of the interpretability of AI models, these will have the potential to be used in other currently prevalent pathologies, such as cardiovascular, neurological or psychiatric diseases. The developed AI-powered tools may be used as clinical support systems for complex diagnosis, and contribute to new diagnostic approaches based on telemedicine or second opinion.

To further ensure future interoperability across repositories of the same nature, the CHAIMELEON is taking part in the AI for Health Imaging (AI4HI) league, a collaborative network of similar projects funded under the same topic. The main goal to this collaboration is to ensure the long-term sustainability of these kind of repositories, and to promote cooperation and data sharing among users.

Further information on the main project objectives, partners and contributors, work progress and latest updates on project results can be found on the project website (chaimeleon.eu) and social media platforms.

## Data Availability Statement

The data collected for this project will be real world patient data, collected retrospectively through the routine delivery of healthcare with no enrollment conditions. This is a purely observational, non-interventional study. Requests to access these datasets should be directed to LM, marti_lui@gva.es.

## Ethics Statement

The studies involving human participants were reviewed and approved by Comité de Ética de la Investigación con Medicamentos (CEIM), Hospital Universitario y Politécnico La Fe. Written informed consent for participation was not required for this study in accordance with the national legislation and the institutional requirements.

## Author Contributions

AM and LM wrote the manuscript. AS and MA conceived the project idea and wrote the proposal for funding with the support of LM. The rest of authors contributed to the conception of the project idea and the project proposal preparation.

## Funding

CHAIMELEON has been funded by as a Horizon 2020 project (RIA, topic DT-TDS-05-2020-AI for Health Imaging; call SC1-FA-DTS-2019-1, under Grant Agreement No. 952172).

## Conflict of Interest

Authors AS, MA were employed by Matical Innovation SL. MM was employed by GE Healthcare. KS was employed by Medexprim. SF way employed by Bahia Software S.L.U. AA-B way employed by QUIBIM SL.

The remaining authors declare that the research was conducted in the absence of any commercial or financial relationships that could be construed as a potential conflict of interest.

The reviewer KL declared a past co-authorship with several of the authors LM, PL to the handling editor.

## Publisher’s Note

All claims expressed in this article are solely those of the authors and do not necessarily represent those of their affiliated organizations, or those of the publisher, the editors and the reviewers. Any product that may be evaluated in this article, or claim that may be made by its manufacturer, is not guaranteed or endorsed by the publisher.
