# Supplementary material for: CHAIMELEON Project: Creation of a Pan-European Repository of Health Imaging Data for the Development of AI-Powered Cancer Management Tools
Source: Front Oncol. 2022 Feb 24;12:742701. doi: 10.3389/fonc.2022.742701 (PMC8913333; doi:10.3389/fonc.2022.742701)
Supplement: Supplementary file 1 [file DataSheet_1.docx]

Supplementary Material

**Dataset submission process and quality checks**

To ensure that the repository offers only quality datasets, the following submission processes are being implemented:

1. To submit a dataset, the contributor data must follow quality assurance tests, as follows:
   - The data is consistent or grouped consistently (e.g., brain MRI, T1-weighted images).
   - Datasets must have a resolution above a certain value, depending on the modality type.
   - Statistical measures and Laplace filters will be used to flag noisy, blurry, and corrupt images to be removed by the contributor.
   - Basic metadata is provided on the dataset as a whole (such as population, lesions) and on each scan (including scan settings).
   - Available annotations are inserted using a standard format common for all of the repository datasets. For example, global annotations will consist of a *csv* file with scan IDs and labels. Point based annotations will consist of a *csv* of scan IDs, coordinates, and labels. Region-based annotations will consist of bit maps (masks) and their respective class types. In all cases, a description will be provided describing the labels and the collection process.
   - All of the above will be performed by the repository's data submission system to simplify the process for the contributor.
2. After submission, grades will be given to datasets based on their quality, namely,
   - User-based scores: Users will be able to up-vote or down-vote datasets based on their experience in terms of ease of use and quality. This will make it easier for other users to find the best datasets for their needs, and encourage the submission of only high-quality datasets.
   - AI-based scores: The repository will automatically train and evaluate 'off-the-shelf' AI models on well-formed and annotated datasets. The performance metrics will then be posted next to each dataset as a baseline for users to note. For example, a low accuracy (e.g., 0-50) may indicate a poor-quality dataset and a very high accuracy (e.g., 99-100) may indicate a non-informative dataset.
   - Security scores: We will use novel detection algorithms to identify any artifacts which are intentionally malicious, aiming to confuse or poison AI models. This will be accomplished using forensic analysis which focuses on identifying adversarial artifacts generated during machine learning attacks. High risk datasets will be automatically removed to be reviewed by the administrator. Medium risk datasets will be published but a warning indicator will be provided to inform the users of any potential risks (e.g., something similar to the green lock-like symbol in a web browser that is next to a secure website URL).
